# Isolated Pancreatic Sarcoidosis Masquerading as Pancreatic Adenocarcinoma: A Case Report

**DOI:** 10.7759/cureus.92547

**Published:** 2025-09-17

**Authors:** Andrew Mims, Sean Rice

**Affiliations:** 1 Gastroenterology, University of Tennessee College of Medicine-Chattanooga, Chattanooga, USA

**Keywords:** granulomatous disease, intestinal sarcoidosis, pancreatic adenocarcinoma, pancreatic disease, whipple procedure

## Abstract

A 50-year-old man with no known medical history presented with a four-year history of epigastric pain and weight loss. Computed tomography (CT) revealed a mass in the head of the pancreas concerning for adenocarcinoma. The patient twice underwent endoscopic ultrasound (EUS) with biopsy, with inconclusive results. Positron emission tomography (PET) scan revealed localized disease, concerning for malignancy. With no definitive diagnosis or treatment options, and after multidisciplinary discussion, the patient underwent a Whipple procedure. Pathology revealed granulomatous inflammation of the pancreas and surrounding lymph nodes, consistent with sarcoidosis. Here, we present a rare case of isolated pancreatic sarcoidosis masquerading as pancreatic malignancy in a patient without a known history of sarcoidosis.

## Introduction

Sarcoidosis is a multisystem, idiopathic disease characterized by granulomatous inflammation. It most commonly affects the lungs; however, any organ can be affected. Symptomatic involvement of the gastrointestinal (GI) tract occurs in less than 1% of cases, and of those, the liver is most commonly involved [1,2]. Pancreatic involvement is exceedingly rare and is usually associated with multiorgan involvement of sarcoidosis. Most cases involving the pancreas are asymptomatic and are discovered on postmortem analysis [3]. The diagnosis of pancreatic sarcoidosis remains difficult, as pancreatic involvement may manifest as direct invasion of the organ, obstruction of the pancreatic or biliary ducts, hepatic lymphadenopathy, or a mimicker of pancreatic adenocarcinoma [4]. Here, we present a rare case of suspected pancreatic malignancy revealed as pancreatic sarcoid after surgical resection, in a patient with no other manifestations of sarcoidosis.

This article was previously presented as a meeting abstract at the 2022 American College of Gastroenterology (ACG) Annual Scientific Meeting on October 24, 2022.

## Case presentation

A 50-year-old male with no known past medical history presented to the gastroenterology clinic reporting 3-4 years of epigastric pain radiating to the back with associated 30-pound weight loss.

**Figure 1:**
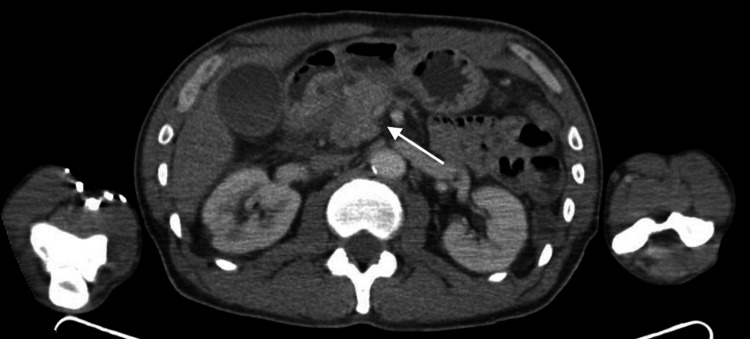
CT scan showing a hyperdense mass-like lesion in the pancreatic head and uncinate process (arrow) CT: computed tomography

**Figure 2:**
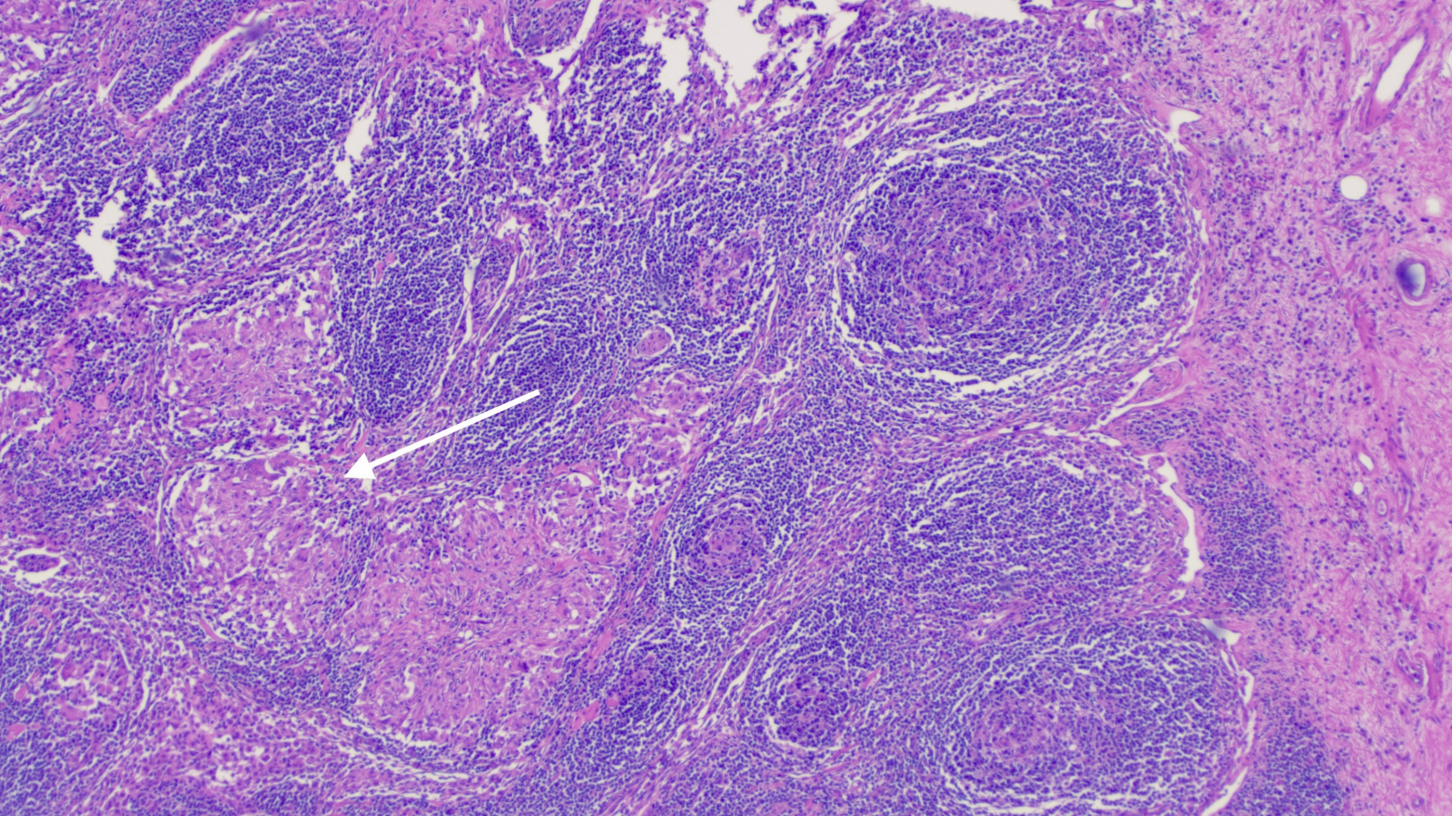
Pathology from peripancreatic lymph node showing lymphohistiocytic inflammatory infiltrate with aggregates of histiocytes and no cellular debris (arrow), consistent with non-necrotizing granulomatous inflammation

He initially presented to an outside emergency department. A computed tomography (CT) scan demonstrated a 2.6-cm hyperdense mass-like lesion with infiltrative margins in the pancreatic head and uncinate process with mild biliary and pancreatic ductal dilation, highly suspicious for pancreatic adenocarcinoma (Figure 1). He underwent urgent endoscopic ultrasound (EUS), which further revealed biliary dilatation with a mass in the head of the pancreas. Pathology from fine needle aspiration (FNA) showed rare atypical cells in a background of chronic inflammation, and a neoplastic process could not be excluded. Stains for acid-fast and fungal organisms were negative. With inconclusive results, the patient underwent repeat EUS, which revealed an irregular mass in the pancreatic head. Pathology from the repeat EUS revealed chronic inflammation and was once again negative for malignancy. Follow-up positron emission tomography (PET) scan, however, revealed a lesion in the pancreatic head that had mild F-fluorodeoxyglucose (FDG) uptake suspicious for malignancy. Of note, IgG4 levels were normal. The case was presented at the multidisciplinary tumor board. Given the inconclusive results with limited treatment options for ongoing debilitating abdominal pain and the possibility of malignancy, the patient underwent a Whipple procedure without complication. Pathology was fortunately negative for malignancy but revealed granulomatous inflammation in the pancreas, with several lymph nodes of the pancreas, bile duct, and liver demonstrating granulomatous inflammation (Figure 2), findings suggestive of sarcoidosis. The postoperative course was uncomplicated. The patient was established with rheumatology and was started on prednisone with clinical improvement. At follow-up, the patient had no evident additional manifestations of sarcoidosis.

## Discussion

Sarcoidosis is a multisystem disease that most commonly affects the lungs, followed by the skin, eyes, joints, heart, and central nervous system. Gastrointestinal involvement most commonly involves the liver, followed by the stomach, and rarely finds its way to the pancreas [5,6]. Pancreatic involvement can manifest in many ways, including mimicking pancreatic adenocarcinoma. While pancreatic involvement of sarcoidosis is common, there have only been a few reported cases of isolated pancreatic sarcoidosis, which makes this case particularly interesting.

This patient presented with intractable abdominal pain with concern for a mass on imaging. He underwent two separate EUS procedures with no evidence of malignancy. These biopsies also revealed no evidence of non-caseating granulomas or other signs of sarcoidosis, as has been seen in other cases of isolated pancreatic sarcoidosis [7]. A follow-up PET scan was concerning for pancreatic malignancy. This provided a difficult clinical circumstance. This patient suffered from debilitating abdominal pain with no definitive diagnosis and thus no definitive treatment option. Furthermore, prompt diagnosis and treatment would be required if this suspicious mass indeed represented malignancy. Ultimately, after multidisciplinary collaboration, the patient underwent a Whipple procedure with relief of symptoms and a situationally fortunate diagnosis of sarcoidosis.

## Conclusions

Given that pancreatic cancer typically portends a poor prognosis, there is greater urgency in diagnosis and treatment for suspicious pancreatic masses. A high degree of clinical suspicion is necessary to consider sarcoidosis in this setting, with the understanding that isolated pancreatic sarcoidosis is exceedingly rare compared to pancreatic malignancy. In pancreatic masses with no clear diagnosis on biopsy, further investigation for other signs of sarcoidosis should be considered.
